# Neutrophil Extracellular Traps of *Cynoglossus semilaevis*: Production Characteristics and Antibacterial Effect

**DOI:** 10.3389/fimmu.2017.00290

**Published:** 2017-03-22

**Authors:** Ming-li Zhao, Heng Chi, Li Sun

**Affiliations:** ^1^Key Laboratory of Experimental Marine Biology, Institute of Oceanology, Chinese Academy of Sciences, Qingdao, China; ^2^Laboratory for Marine Biology and Biotechnology, Qingdao National Laboratory for Marine Science and Technology, Qingdao, China; ^3^University of Chinese Academy of Sciences, Beijing, China

**Keywords:** neutrophil extracellular trap, NETosis, *Cynoglossus semilaevis*, antibacterial, innate immune defense

## Abstract

Neutrophil extracellular traps (NETs) are structures released by neutrophils as a cellular immune defense against microbial invasion. The process of NETs generation, netosis (NETosis), can take place *via* either a suicidal mechanism, during which the NETs-releasing cells became dead, or a “live” mechanism, during which the NETs-releasing cells remain vital. NETosis has been studied intensively in mammals in recent years, but very little is known about the NETosis in fish. In this study, we examined NETosis in tongue sole (*Cynoglossus semilaevis*), a species of teleost with important economic values. We found that following stimulation with phorbol 12-myristate 13-acetate (PMA) and three common fish bacterial pathogens, abundant NETs structures were released by neutrophils that were most likely in a live state. The released NETs captured, but did not kill, the bacterial pathogens; however, the replication of extracellular, but not intracellular, pathogens was inhibited by NETs to significant extents. Reactive oxygen species (ROS), nitric oxide (NO), and myeloperoxidase (MPO) production were observed to be enhanced in NETosing neutrophils, and blocking the production of these factors by inhibitors significantly decreased NETs production induced by PMA and all three bacteria. Taken together, these results indicate for the first time that in teleost there exists a non-cell death pathway of NETosis that produces NETs with antibacterial effects in a ROS-, NO-, and MPO-dependent manner.

## Introduction

Neutrophils are professional phagocytes that serve as the first line of defense against invading pathogens, thus playing a pivotal role in innate immune defense (1). Neutrophils kill microorganisms by phagocytosis and oxidative burst. In 2004, Brinkmann and colleagues reported NETosis, a new cell death pathway in human neutrophils that was distinct from apoptosis and necrosis and relied on a structure called neutrophil extracellular traps (NETs) (2, 3). NETs are web-like structures composed of extracellular DNA, histones, and antimicrobial proteolytic enzymes in the granules of neutrophils, such as neutrophil elastase and myeloperoxidase (MPO) (4). NETs have been reported to capture and immobilize bacteria; however, there is much controversy about whether NETs can directly kill bacteria (5).

In mammals, NETs are released from neutrophils in response to a wide spectrum of pro- and anti-inflammatory stimuli, such as phorbol 12-myristate 13-acetate (PMA), lipopolysaccharides, bacteria, fungi, viruses, and parasites (6). NETs were detected in mammals like mice (7), humans, rabbit (3), and dogs (8), as well as in chicken and marine animals (9, 10). However, reports from different laboratories often vary with respect to the timing and effectiveness of NETs production in response to different stimuli, which suggests that there exist more than one mechanism of NETs formation (11). Evidences have indicated that the formation of NETs requires the generation of reactive oxygen species (ROS) and nitric oxide (NO) (12–14). In addition, MPO, one of the most abundant proteins in neutrophils, is also a constituent of NETs (3, 15), and it has been reported that neutrophils from patients who lack the ability to produce MPO fail to produce NETs (16).

Compared to NETs studies in mammalian models, studies of NETs in fish are few. Although NETs-like structures have been observed in teleost species including turbot, fathead minnows, carp, and zebrafish (17–20), the underlying mechanism regulating NETosis and the functionality of NETs in fish are essentially unknown. Tongue sole (*Cynoglossus semilaevis*) is a flatfish with important economic values in China. In this study, we investigated the characteristics of NETs production by tongue sole neutrophils, with an emphasis on the effect of ROS, NO, and MPO. We also examined the antimicrobial effect of NETs against common fish bacterial pathogens.

## Materials and Methods

### Ethics

Experiments involving live animals were conducted in accordance with the “Regulations for the Administration of Affairs Concerning Experimental Animals” promulgated by the State Science and Technology Commission of Shandong Province. The study was approved by the Ethics Committee of Institute of Oceanology, Chinese Academy of Sciences.

### Neutrophil Isolation

Clinically healthy tongue sole (average 250 g) were purchased from a local fish farm. Isolation of neutrophils was performed as reported previously (17, 21, 22) with modifications as follows. Head kidney was aseptically collected from tongue sole and placed into a 50 ml test tube containing 30 ml of Hank’s balanced salt solution with Ca^2+^ and Mg^2+^ but without phenol red (HBSS) (Mediatech-CellGro, AK, USA). The tissue was processed by being passed through a 100 μm nylon Falcon cell strainer (BD Falcon, Lexington, KY, USA). The cells were isolated using 51% percoll (GE Healthcare, Uppsala, Sweden), with the buffy coat being removed. The remaining red blood cell/granulocyte pellet was collected, and the red blood cells were separated using a specific gravity of 61% percoll. The granulocytes/macrophages were collected and pooled. The cells were washed twice in HBSS and seeded into 25 cm^3^ polystyrene cell culture flasks (Costar, Tewksbury, MA, USA) containing L15 medium (HyClone, Logan, UT, USA) supplemented with 2% fetal bovine serum (HyClone, Logan, UT, USA) and cultured at 22°C with 5% CO_2_ for overnight. The non-adherent neutrophil-like cells were harvested, and a portion of the cells were used for total cell count, and cytochemical staining as reported previously (17). As shown in Figure S1 in Supplementary Material, most of the prepared cells were neutrophils.

### Microscopy

The inducers of NETosis used in this study were PMA and three fish bacterial pathogens. PMA was used because it is known to be a very effective inducer of NETosis in mammalian models (2), and therefore served in this study as a positive control for the three bacteria pathogens. Microscopic observation of NETs was performed as reported previously (17) with modifications. Briefly, the fish bacterial pathogens *Pseudomonas fluorescens, Vibrio harveyi*, and *Edwardsiella tarda* (23–25) were cultured in Luria–Bertani broth (LB) medium at 28°C to an OD_600_ of 0.8; the cells were collected by centrifugation and washed with PBS. Neutrophils (~10^6^) were seeded onto a glass coverslip that had already been treated with 0.001% polylysine (Sigma, St. Louis, MO, USA) and placed in a 12-well cell culture plate. The cells were allowed to settle for 2 h and then treated with PMA (1 μg ml^−1^) (Sigma, St. Louis, MO, USA), *P. fluorescens* (1 × 10^6^ CFU), *V. harveyi* (1 × 10^6^ CFU), or *E. tarda* (1 × 10^6^ CFU) at 22°C for 2 h. The control cells were left untreated. For fluorescent microscopy, Sytox Green was added to the cells, and after incubation for 5 min, the cells were washed three times with PBS. Then the cells were fixed with 4% paraformaldehyde (Sigma, St. Louis, MO, USA) for 25 min and stained with 4′,6-diamidino-2-phenylindole (DAPI) according to the instructions of the manufacturer (Bioss, Beijing, China). Scanning electron microscopy (SEM) was performed as reported (10).

### Survival of NETs-Trapped Bacteria

The survival of NETs-entrapped bacteria was examined as reported previously (17). Briefly, bacterial cells were cultured in LB medium to an OD_600_ of 0.8 and harvested by centrifugation. The bacterial cells were washed with PBS for three times and resuspended in PBS. Neutrophils (2 × 10^5^ cells/well in a 200 μl volume) were seeded in 96-well cell culture plates and allowed to adhere for 60 min at 22°C. The cells were then stimulated with 1 μg ml^−1^ PMA for 2 h and centrifuged at 400 *g* for 5 min, and 150 μl of supernatant was discarded. Then, 50 μl L15 medium containing or not containing 100 U ml^−1^ DNase I was added to the cells. The cells were maintained at 22°C for 20 min, then cytochalasin D (20 μg ml^−1^) (Invitrogen, Carlsbad, CA, USA) and 2,000 CFU bacteria (*P. fluorescens, V. harveyi*, or *E. tarda*) were added to the plates. The plates were centrifuged at 800 *g* for 10 min to allow intimate contact of the bacteria with NETs/neutrophils. The plates were then incubated at 22°C for 2, 4, 6, and 8 h. After incubation, the content of each well (bacteria plus neutrophils and NETs) was taken out and serially diluted, and the dilutions were plated on LB agar plates. The plates were incubated at 28°C for 24 h, and the colonies that emerged on the plates were counted. The genetic nature of the colonies was verified by PCR and sequence analysis of the PCR products.

### Quantification of NETs

To quantify NETs, the method of Parker et al. (11) was adopted. Briefly, neutrophils (5 × 10^6^ cells/well in a 200 μl volume) were suspended in HBSS (Mediatech-CellGro, USA) and seeded in a black 96-well plate (Cayman Chemical, Ann Arbor, MI, USA). The cells were treated with PMA (1 μg ml^−1^), *P. fluorescens* (1 × 10^6^ CFU), *V. harveyi* (1 × 10^6^ CFU), or *E. tarda* (1 × 10^6^ CFU) for 1, 2, 3, or 4 h. The control cells were untreated. After treatment, the membrane-impermeable DNA-binding dye, Sytox Green (5 μM), was added to the cells, followed by incubation for 5 min. Fluorescence was then quantified as relative fluorescence units (RFU) at 485 nm excitation and 530 nm emission using a fluorescence spectrophotometer (Infinite M1000, Tecan, Switzerland). For NETs inhibition assay, neutrophils were pre-incubated with the following inhibitors for 30 min at 22°C: 100 μM ROS scavenger Trolox (Sigma, St. Louis, MO, USA), 1 mM nitric oxide (NO) inhibitor N(G)-nitro-L-arginine methylester L-NAME (Sigma, St. Louis, MO, USA), or 100 μM MPO inhibitor 4-aminobenzoic acid hydrazide (ABAH) (Calbiochem, San Diego, CA, USA). NETs production and measurements were then performed as above.

### Measurement of ROS, NO, and MPO

To measure ROS and NO production, the methods described by Patel et al. and Lim et al. (26, 27) were adopted, respectively. Briefly, neutrophils were suspended in HBSS and seeded in a black 96-well plate (5 × 10^6^ cells/well). The cells were incubated with 10 μM 2′,7′-dichlorofluorescein diacetate (DCFH-DA) (Sigma, USA) at 22°C for 20 min for ROS quantification and with 5 μM 4,5-diaminofluorescein diacetate (DAF-2DA) (Sigma, USA) for NO quantification, respectively. The plates were centrifuged for 10 min at 400 *g*, and the supernatant was replaced with fresh HBSS containing PMA (1 μg ml^−1^), *P. fluorescens* (1 × 10^6^ CFU), *V. harveyi* (1 × 10^6^ CFU), or *E. tarda* (1 × 10^6^ CFU). The control cells were untreated. The plates were incubated at 22°C for 20, 40, 80, 100, and 120 min. After incubation, ROS and NO were measured as RFU at 485 nm excitation and 525 nm emission and at 495 nm excitation and 515 nm emission, respectively, using a fluorescence spectrophotometer (Infinite M1000, Tecan, Switzerland).

Myeloperoxidase release was measured as reported previously (18). Briefly, neutrophils were suspended in HBSS and seeded in a 96-well plate (5 × 10^6^ cells/well). The cells were treated with PMA (1 μg ml^−1^), *P. fluorescens* (1 × 10^6^ CFU), *V. harveyi* (1 × 10^6^ CFU), or *E. tarda* (1 × 10^6^ CFU). The control cells were untreated. The cells were incubated at 22°C for 60 and 120 min, and 50 μl of 3,3′,5,5′-tetramethylbenzidine hydrochloride (TMB) (Sigma, USA) was added to the cells, followed by immediately adding 50 μl of hydrogen peroxide. The color change reaction was allowed to proceed for 3 min, and then 50 μl 2 M sulfuric acid was added to stop the reaction. The plates were centrifuged at 400 *g* for 15 min, and 200 μl of the supernatant from each well was transferred to another plate and the optical density in each well was determined at 405 nm.

### Statistical Analysis

All experiments were performed three times, and statistical analyses were performed using analysis of variance with SPSS 17.0 software (SPSS Inc., Chicago, IL, USA). Statistical significance was determined with Student’s *t*-test. In all cases, significance was defined as *P* < 0.05.

## Results

### Production of NETs by Live Neutrophils

**Figure 1:**
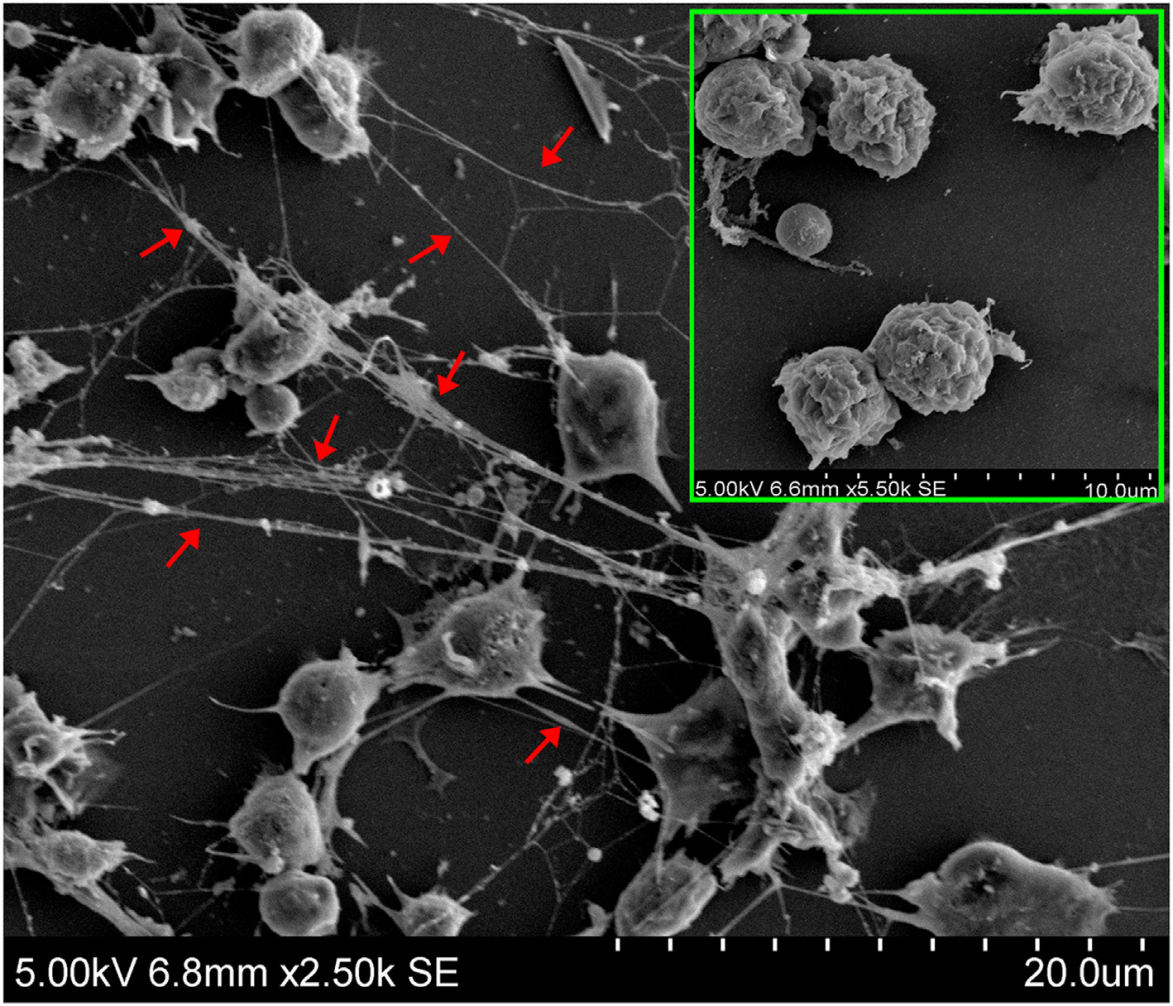
**Production of neutrophil extracellular traps (NETs) by neutrophils in response to phorbol 12-myristate 13-acetate (PMA) treatment**. Tongue sole neutrophils were treated with or without (boxed image) PMA and observed with a scanning electron microscope. Arrows indicate NETs.

**Figure 2:**
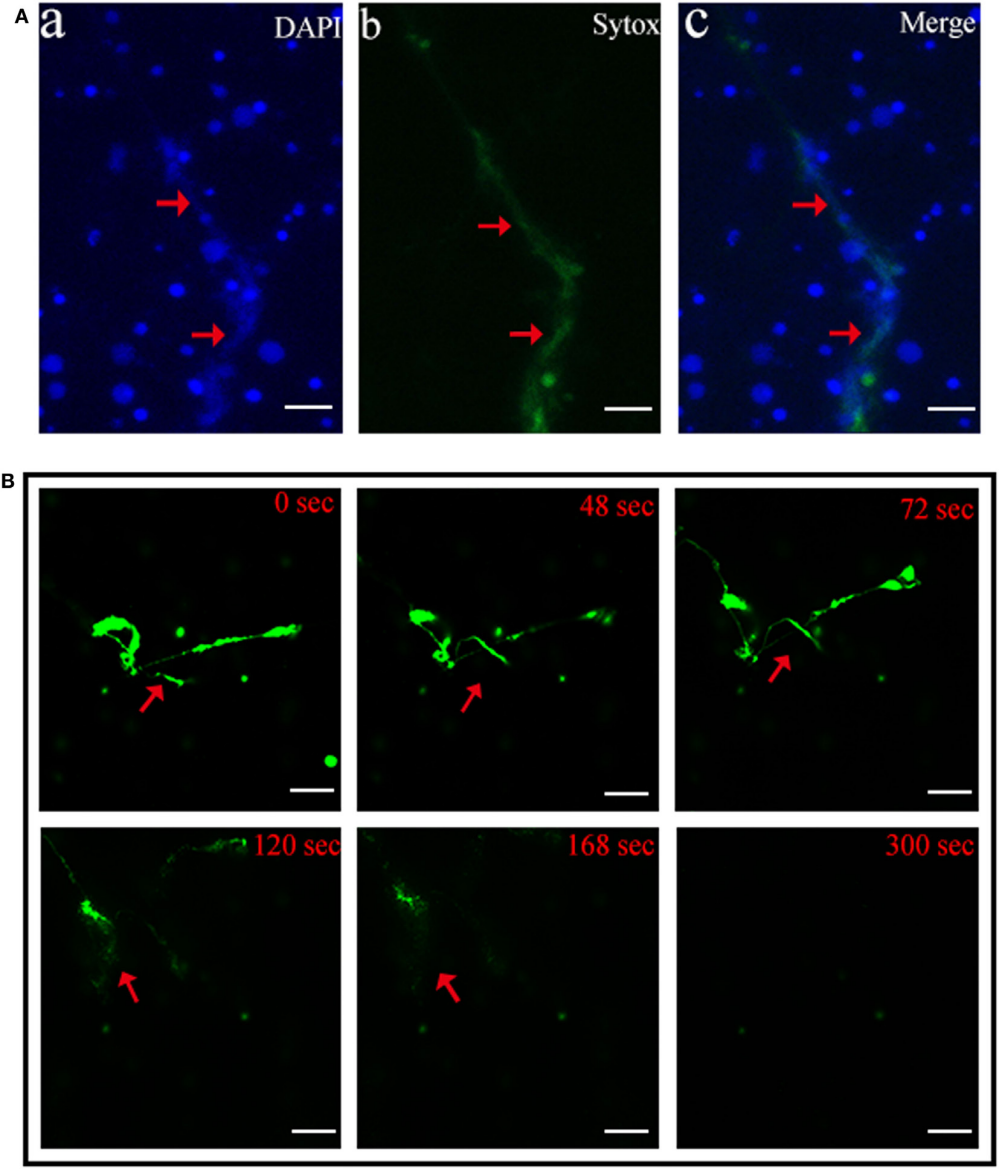
**Production of neutrophil extracellular traps (NETs) by live neutrophils**. **(A)** Tongue sole neutrophils were treated with phorbol 12-myristate 13-acetate (PMA) and stained with Sytox Green and DAPI. The cells were then observed with a fluorescence microscope. **(B)** PMA-treated neutrophils were stained with Sytox Green and incubated with DNase I for different times. The cells were observed as above. Arrows indicate NETs. Bar, 20 μm.

Scanning electron microscopy showed that when tongue sole kidney neutrophils were treated with PMA, abundant NETs structures were produced, which contained long stretches of fibers dotted with spherical objects similar to the 25–50 nm globular protein domains observed in mammalian NETs (3) (Figure 1). To examine whether the NETs-producing neutrophils were alive, the cells were stained with Sytox Green, which is impermeable to live cells, and DAPI after PMA treatment. The results indicated that DAPI was associated with both NETs and the cells, whereas Sytox Green was excluded from the cells (Figure 2A), suggesting that NETs were produced by live cells. Consistent with this observation, when PMA-induced NETs was incubated with DNase I, the NETs structure gradually disappeared as the incubation time increased (Figure 2B; Video S1 in Supplementary Material), suggesting that DNA was an essential component of NETs.

### NETosis Induced by Fish Pathogens

**Figure 3:**
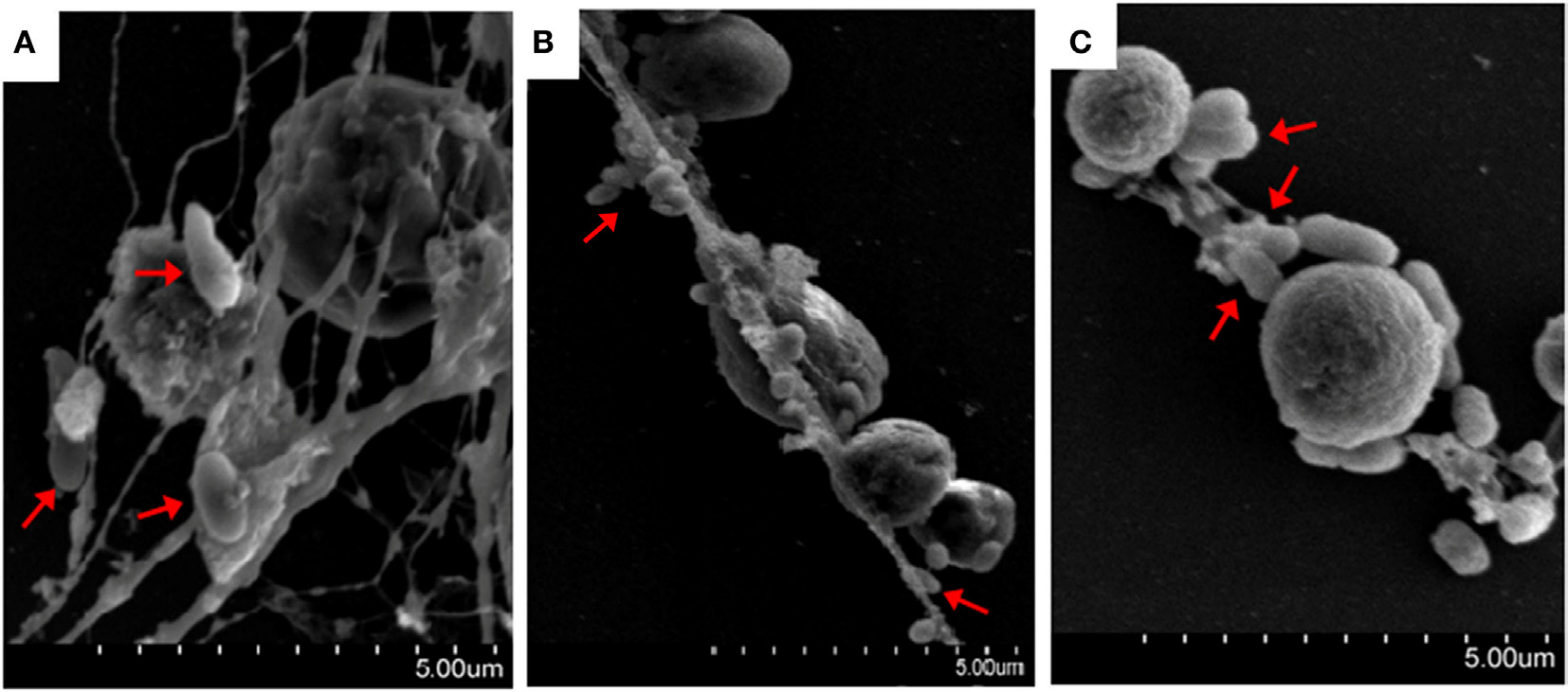
**Bacteria-induced production of neutrophil extracellular traps (NETs)**. Tongue sole neutrophils were treated with *Pseudomonas fluorescens* **(A)**, *Vibrio harveyi* **(B)**, and *Edwardsiella tarda* **(C)**, and the cells were observed with a scanning electron microscope. Arrows indicate NETs-trapped bacteria.

**Figure 4:**
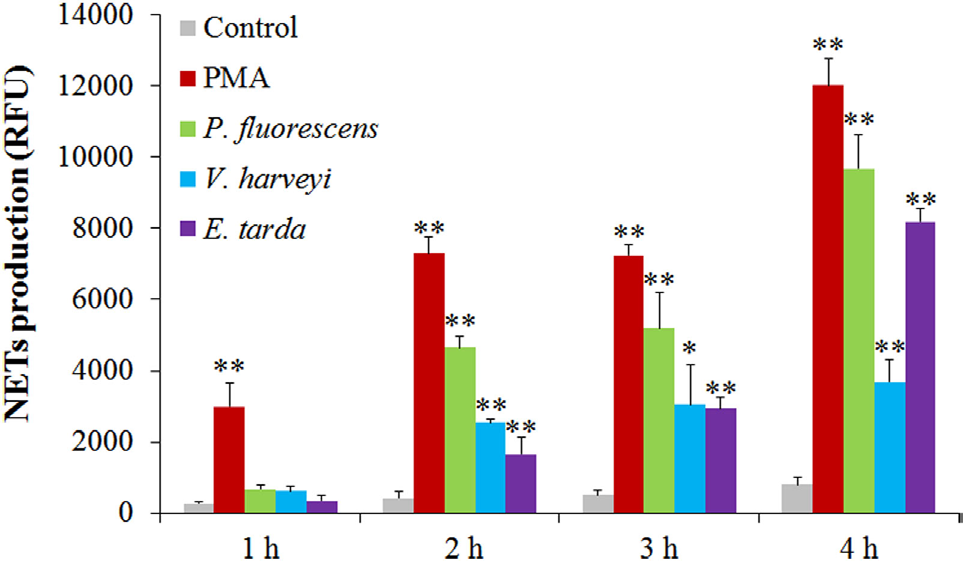
**Time-course production of neutrophil extracellular traps (NETs) by neutrophils in response to various treatments**. Tongue sole neutrophils were treated with or without (control) phorbol 12-myristate 13-acetate (PMA), *Pseudomonas fluorescens, Vibrio harveyi*, and *Edwardsiella tarda* for various hours, and NETs production was determined. The experiment was performed three times, and the results are shown as means ± SEM. **P < 0.01; *P < 0.05.

The bacteria *E. tarda, P. fluorescens*, and *V. harveyi* are common pathogens to marine fish including tongue sole. To investigate their effects on NETosis, tongue sole neutrophils were incubated with each of these bacteria, and NETs production was subsequently determined microscopically. The results showed that SEM detected NETs formation in all groups of neutrophils treated with the bacteria, and that the produced NETs were able to trap the bacterial cells (Figure 3). Quantitative analysis revealed that bacteria-induced NETs production in a time-dependent manner, with the amounts of NETs increasing from 1 to 4 h after bacterial treatment (Figure 4).

### NETs Entrapment on Bacterial Survival

**Figure 5:**
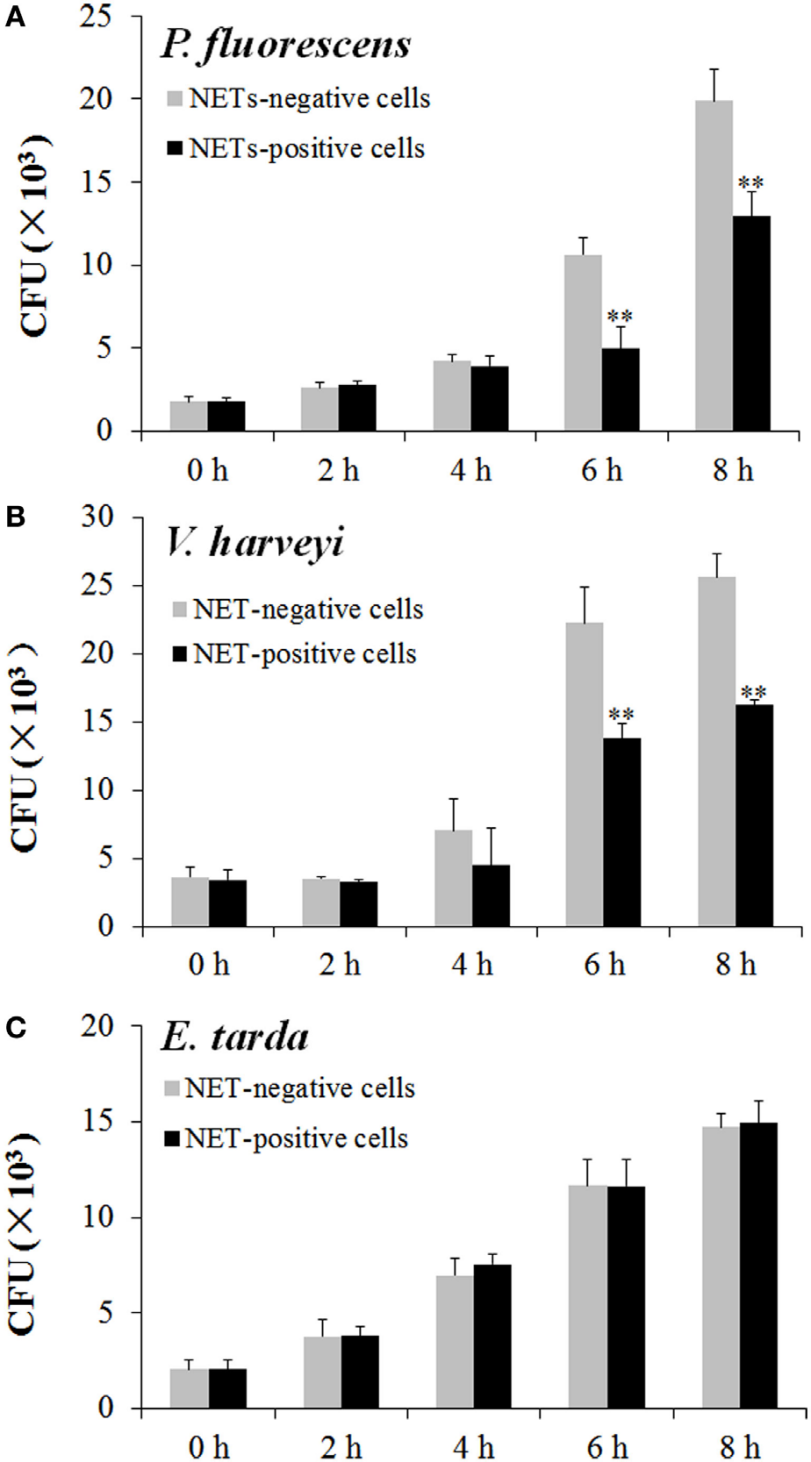
**Multiplication of neutrophil extracellular traps (NETs)-trapped bacteria**. Neutrophils were stimulated with phorbol 12-myristate 13-acetate for NETs production (NETs-positive cells), and a portion of the cells were then treated with DNase I to degrade NETs (NETs-negative cells). NETs-positive and NETs-negative cells were incubated with *Pseudomonas fluorescens* **(A)**, *Vibrio harveyi* **(B)**, and *Edwardsiella tarda* **(C)** for different hours, and bacterial numbers were determined by plate count. The experiment was performed three times, and the results are shown as means ± SEM. **P < 0.01.

To examine the fate of the bacteria trapped by NETs, NETs-positive neutrophils and NETs-negative neutrophils were incubated with *E. tarda, P. fluorescens*, and *V. harveyi* for 0, 2, 4, 6, or 8 h, and viable bacteria were recovered at each time point. The results showed that for *P. fluorescens* and *V. harveyi*, the numbers of viable bacteria after 2 and 4 h incubation with NETs-positive neutrophils were comparable to those after incubation with NETs-negative neutrophils; however, after 6 and 8 h incubation, the bacterial recoveries from NETs-positive neutrophils were significantly lower than those from NETs-negative neutrophils (Figures 5A,B). By contrast, for *E. tarda*, bacterial recoveries from NETs-positive neutrophils were similar to those from NETs-negative neutrophils at all time points (Figure 5C).

### ROS, NO, and MPO Production in Bacteria-Treated Neutrophils

**Figure 6:**
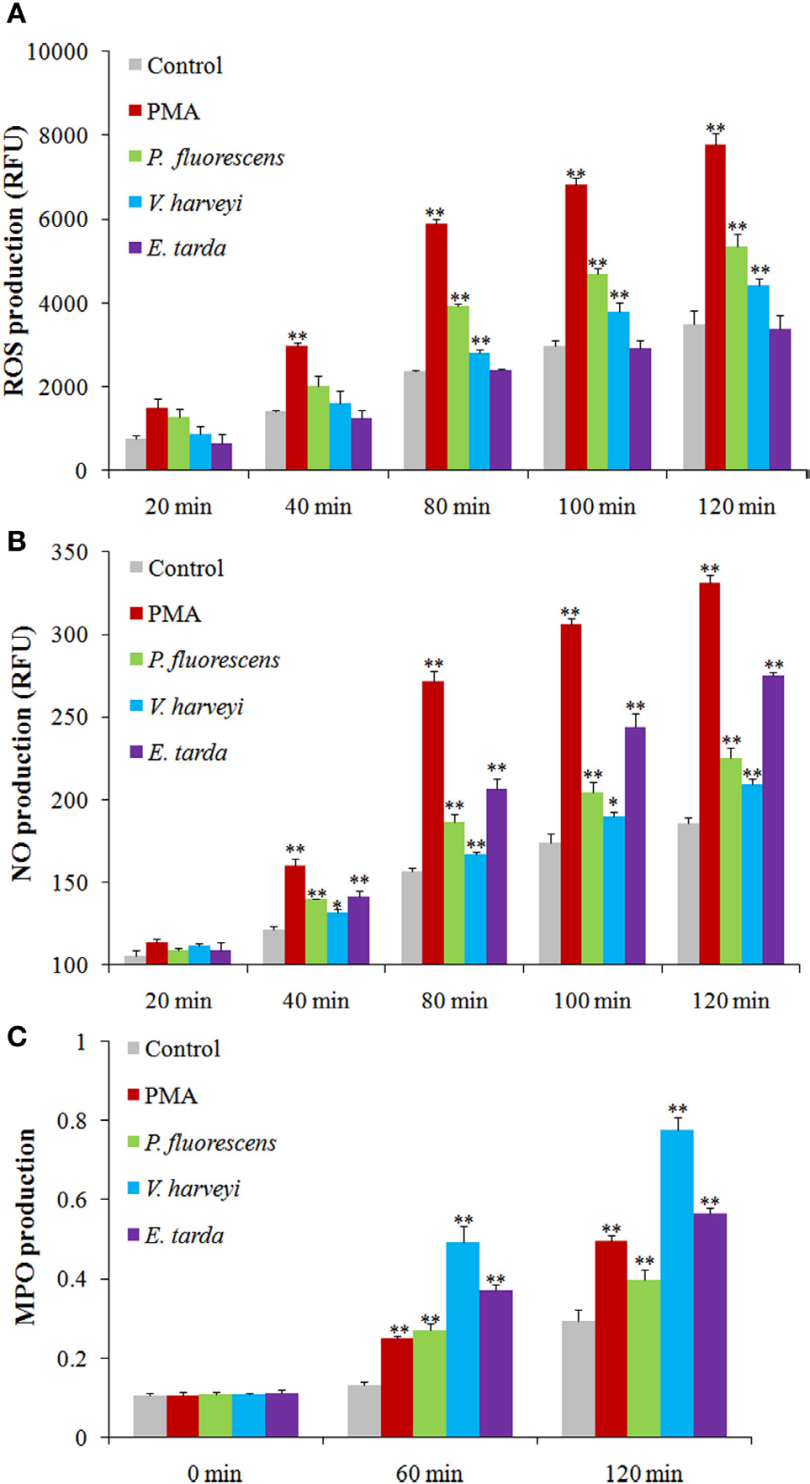
**Production of reactive oxygen species (ROS), NO, and myeloperoxidase (MPO) in neutrophils after different treatments**. Tongue sole neutrophils were treated with or without (control) phorbol 12-myristate 13-acetate (PMA), *Pseudomonas fluorescens, Vibrio harveyi*, and *Edwardsiella tarda* for different times, and ROS **(A)**, NO **(B)**, and MPO **(C)** productions were determined. The experiment was performed three times, and the results are shown as means ± SEM. **P < 0.01; *P < 0.05.

Reactive oxygen species analysis showed that in neutrophils treated with PMA, *P. fluorescens*, and *V. harveyi*, ROS levels increased with time, whereas in neutrophils treated with *E. tarda*, no apparent change in ROS was observed (Figure 6A). For NO and MPO, their productions were enhanced significantly in a time-dependent fashion in neutrophils treated with all tested stimulants, i.e., PMA, *E. tarda, P. fluorescens*, and *V. harveyi*, with *E. tarda* being the strongest inducer among all bacteria for NO production (Figure 6B), and *V. harveyi* for MPO production (Figure 6C).

### Essentialness of ROS, NO, and MPO to Bacteria-Induced NETosis

**Figure 7:**
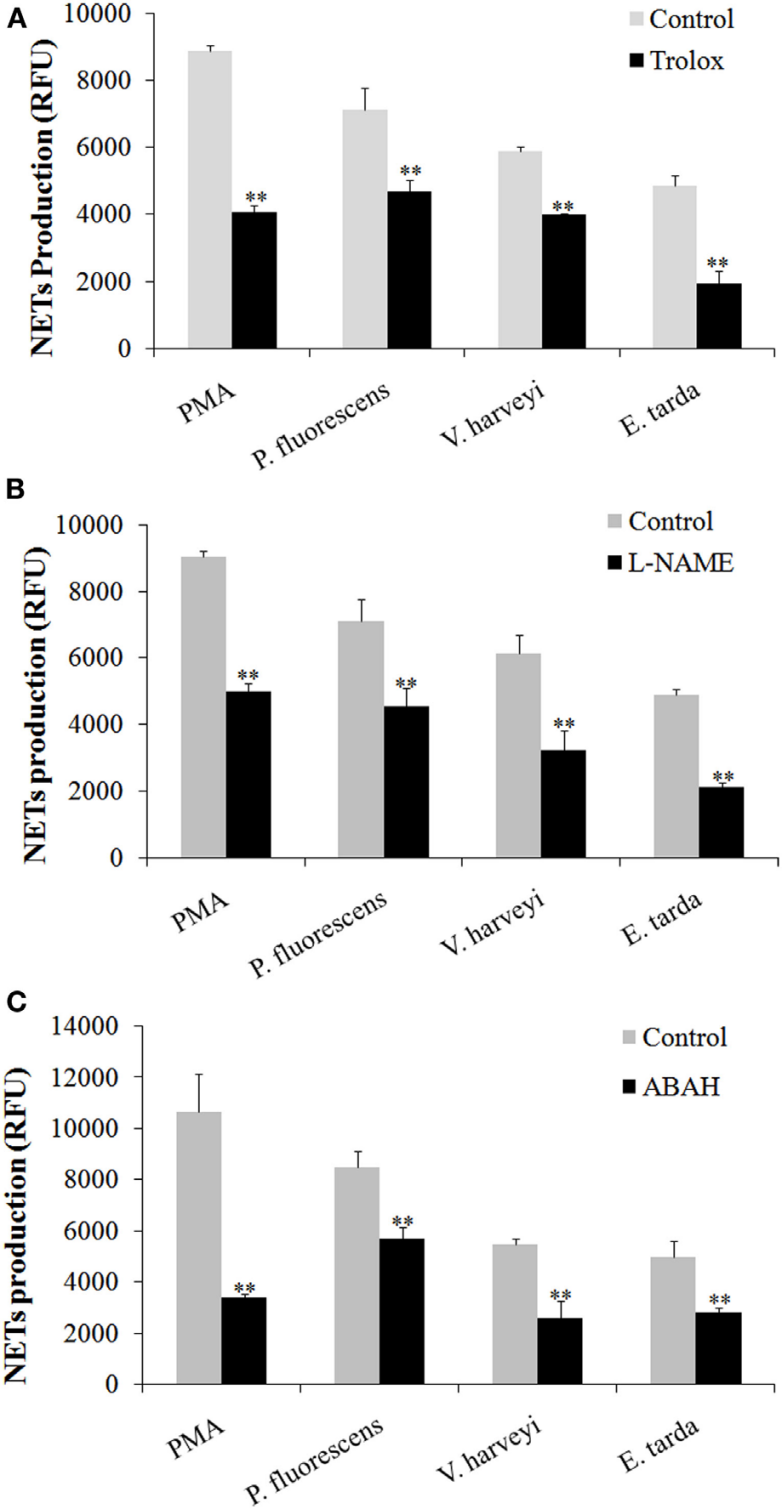
**Production of neutrophil extracellular traps (NETs) in response to various inhibitors**. Tongue sole neutrophils were treated with phorbol 12-myristate 13-acetate (PMA), *Edwardsiella tarda, Pseudomonas fluorescens*, or *Vibrio harveyi* in the presence or absence (control) of Trolox **(A)**, L-NAME **(B)**, and ABAH **(C)**, and NETs production was determined. The experiment was performed three times, and the results are shown as means ± SEM. **P < 0.01.

Given the above observation, we wondered whether ROS, NO, and MPO production was required for NETosis. To investigate this question, neutrophils were treated with PMA, *E. tarda, P. fluorescens*, and *V. harveyi* in the presence of the inhibitor against the production/accumulation of ROS, NO, or MPO, and the amount of NETs produced by the cells was subsequently determined. The results showed that the presence of Trolox, L-NAME, and ABAH, which are inhibitors of ROS, NO, and MPO, respectively, caused significant NETs reduction in all groups of neutrophils (Figure 7).

## Discussion

In mammals, NTEs are released by neutrophils in response to treatment with various stimulants including PMA, microorganisms, and cytokines (2, 3, 28, 29). In the current study, we found that after treatment with PMA, tongue sole neutrophils released abundant NETs structures. Brinkmann et al. have reported that in mammalian NETs, the diameter of single DNA stretches was 15–17 nm, and the DNA stretches were punctuated with globular protein domains ranging from 25 to 50 nm (3). In tongue sole NETs, we also observed smooth stretches of DNA fibers and intermittent globular complexes. Double-staining with the DNA specific fluorescent dyes Sytox Green and DAPI showed that while DAPI stained both the extracellular DNA in NETs and the nuclei of the cells, Sytox Green was associated only with the extracellular DNA in NETs, indicating that NETs were produced by living neutrophils. These results support the idea of vital NETosis, a process during which the cells release NETs while still maintaining their natural defense capacities as live cells (30).

*Edwardsiella tarda, V. harveyi*, and *P. fluorescens* are among the most common aquaculture pathogens with a wide host range including a large number of fish and shellfish. Of these bacteria, *E. tarda* is an intracellular pathogen known to be able to evade host immunity and replicate inside host phagocytes (31, 32). In this study, we found that all three bacteria triggered apparent NETosis in a time-dependent manner, and that the amounts of NETs formed varied after different bacterial treatments, suggesting that these pathogens differed in their NETs-inducing capacities. NETs have been reported to immobilize microbes since they were first discovered (3); however, the ability of NETs to kill microbes appears to differ under different conditions. For instance, *Streptococcus pneumoniae* and *Streptococcus aureus* were reported to be captured, but not killed, by mammalian NETs (7, 33, 34), whereas *Streptococcus flexneri* and *Candida albicans* were directly killed by NETs (3, 15). In fish, our recent study demonstrated that turbot NETs were able to kill *Escherichia coli* but not *P. fluorescens* (17). In the case of tongue sole NETs, we observed entrapment of *P. fluorescens, V. harveyi*, and *E. tarda* by NETs. Plate count analysis showed that after NETs entrapment, the bacterial numbers of *V. harveyi* and *P. fluorescens* increased with time but at a rate significantly slower than that of the control bacteria, suggesting that these entrapped bacteria were still alive and capable of replication though in a significantly slower manner compared to the control bacteria. These results indicated that NETs immobilization did not kill *V. harveyi* and *P. fluorescens* but did interfere with the replication of these bacteria. In contrast to *V. harveyi* and *P. fluorescens*, entrapped *E. tarda* increased in number in a manner similar to that of the control cells, suggesting that NETs entrapment had no apparent impact on *E. tarda* as far as multiplication is concerned, which implies that *E. tarda* possesses a certain mechanism that enables the pathogen to resist the antimicrobial effect of NETs.

In mammals, evidences have shown that NETosis requires the generation of ROS by NADPH oxidase. Neutrophils from patients with mutations in NADPH oxidase and knockout mice that lacked functional NADPH oxidase failed to produce NETs in response to PMA (12, 13); NETs formation upon activation with bacteria was also impaired after pharmacological inhibition of respiratory burst with NADPH oxidase inhibitor (2). The underlying mechanism is that ROS is able to activate PAD4, which in turn mediates the citrullination of histone and results in chromatin decondensation, which is an essential of NETosis (5). Besides ROS, NO is another key player in NET formation both in human and mouse neutrophils (26, 27). It has shown that the NO mediates NETs release through free radical generation involving NADPH oxidase and MPO (27). In our study, we found that the presence of ROS and NO inhibitors significantly decreased NETosis triggered by *V. harveyi* and *P. fluorescens*. These results, together with the observation that both *V. harveyi* and *P. fluorescens* stimulated ROS and NO production in neutrophils, indicated that ROS and NO are important factors for NETosis induced by these pathogens. For *E. tarda*, we found that it enhanced NO production in neutrophils and, in line with this observation, its ability to produce NETs was significantly reduced by L-NAME, suggesting an essential role of NO in NETs formation. Although *E. tarda* did not promote ROS induction, it was affected significantly by ROS inhibitor in the capacity of NETosis. These results suggest that *E. tarda*-triggered NETosis required ROS, likely at a relatively low level, such as that constitutively expressed by the cells.

Myeloperoxidase is known to be a constituent of mammalian NETs (3, 15). MPO is released from azurophilic granule and translocates to the nucleus, where it binds to chromatin and promotes chromatin decondensation, whereby leading to NET release (4). It has been shown that a partial disorder of MPO production resulted in a reduction and delay of NETs formation, and that neutrophils completely deficient in MPO failed to produce NETs in response to PMA and *C. albicans* in humans (16). Parker et al. reported that in human neutrophils, MPO was required for NETosis triggered by PMA, but bacteria-induced NETosis was independent of MPO activity (11). In tongue sole neutrophils, we found that MPO production was enhanced by PMA as well as three bacterial pathogens, and that the presence of MPO inhibitor significantly reduced NETs production caused by PMA, *E. tarda, V. harveyi*, and *P. fluorescens*, indicating an essential role of MPO in NETosis triggered by these inducers. These observations suggest a possible difference in the mechanism of bacteria-induced NETosis between fish and human neutrophils.

In conclusion, we, in this study, demonstrated for the first time that (i) NETs were produced by live neutrophils of tongue sole after chemical and bacterial stimulation, which suggests the existence of a non-cell death pathway of NETosis in fish; (ii) the fish NETs were able to immobilize bacterial pathogens and inhibit the replication of *V. harveyi* and *P. fluorescens* but not the replication of the intracellular pathogen *E. tarda*, which suggests that evasion of NETs-mediated immune response is probably a virulence strategy of *E. tarda*; and (iii) in both bacteria- and PMA-triggered NETosis, ROS, NO, and MPO play a significant role, suggesting a common fundamental NETs production process in tongue sole neutrophils. Taken together, these findings add new insights into the mechanism of NETosis and the functionality of NETs in fish.

## Author Contributions

LS conceived and designed the experiments; M-lZ and HC performed the experiments; M-lZ and HC analyzed the data; M-lZ and LS wrote the manuscript. All authors read and approved the final manuscript.

## Conflict of Interest Statement

The authors declare that the research was conducted in the absence of any commercial or financial relationships that could be construed as a potential conflict of interest.

## Supplementary Material

Figure S1 **Microscopic examination of tongue sole neutrophils**. Neutrophils from tongue sole kidney were stained with potassium iodide–pyronine G and observed with a microscope. The cells with brown granules were neutrophil-like cells, some of which are indicated by arrows. Bar = 10 μM.
