# Supplementary figures and images for: Neutrophil Extracellular Traps of Cynoglossus semilaevis: Production Characteristics and Antibacterial Effect
Source: Front Immunol. 2017 Mar 22;8:290. doi: 10.3389/fimmu.2017.00290 (PMC5360709; doi:10.3389/fimmu.2017.00290)

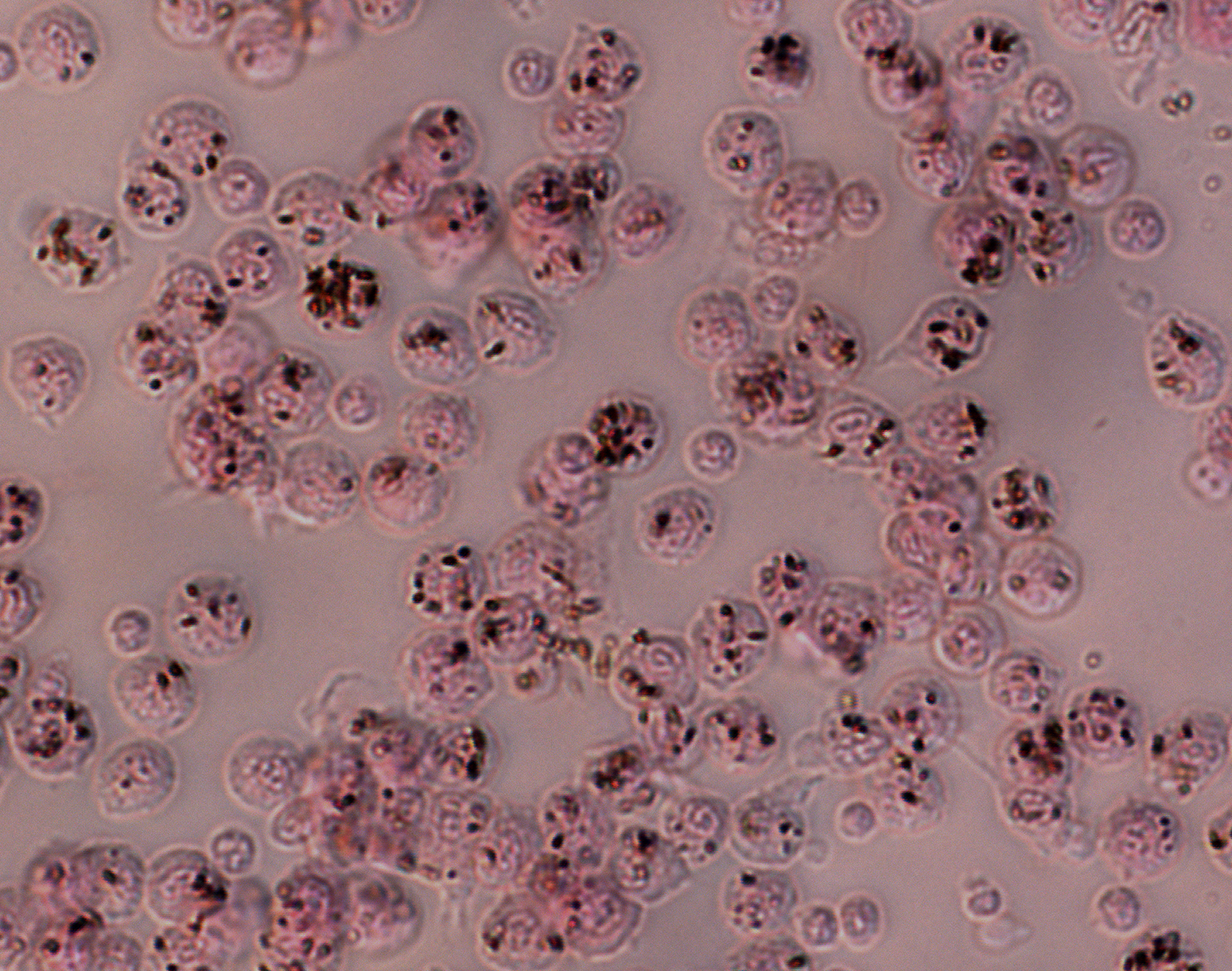

Supplement: Figure S1 — Microscopic examination of tongue sole neutrophils. Neutrophils from tongue sole kidney were stained with potassium iodide–pyronine G and observed with a microscope. The cells with brown granules were neutrophil-like cells, some of which are indicated by arrows. Bar = 10 μM. [file Image_1.TIF]
